# The Role of Diet in Dementia Prevention: Subjective and Objective Diet Scores

**DOI:** 10.1002/alz70860_102850

**Published:** 2025-12-23

**Authors:** Emily A Johnston, Alexandra Nordyke, Joshua Chodosh, Jeannette M Beasley

**Affiliations:** ^1^ NYU Grossman School of Medicine, New York, NY, USA; ^2^ NYU Alzheimer's Disease Research Center, New York, NY, USA

## Abstract

**Background:**

By 2050, it is predicted that 150 million people globally will have Alzheimer's Disease (AD). Current disease modifying therapies are not widely available and nearly 40% of dementias could be prevented or delayed through modifying risk factors. Observational studies show that adherence to the Mediterranean DASH Intervention for Neurodegenerative Delay (MIND) diet is associated with lower risk of cognitive decline and lower cognitive age. The MIND diet is rich in bioactives, including carotenoids, which have antioxidant and anti‐inflammatory properties. Few adults discuss AD‐risk reduction with their healthcare providers and dietary recommendations for AD risk reduction are not a part of routine care.

**Method:**

In this ongoing study, 150 participants of the New York University (NYU) Alzheimer's Disease Research Center (ADRC) are asked to complete a subjective (15‐item MIND diet score) and objective (Veggie Meter) diet assessment at their annual study visit. The Veggie Meter is a reflection spectroscopy device that measures carotenoid levels non‐invasively and correlates with plasma carotenoids. We tested the strength of the relationship between the MIND diet and Veggie Meter scores. Participants receive a report of their scores with resources to learn more.

**Result:**

117 participants have complete data (mean age: 73 ± 7.4). Participants are 73% female; 59% of participants identified as White, 41% as Black or African American, 7% Hispanic, and <1% as Asian. The mean MIND diet score was 7.1 ± 2.1 (max score 15; 47% ± 14 adherence). The mean Veggie Meter score was 339 ± 126 (max score 850). 85% of participants report taking dietary supplements. Preliminary correlations between Veggie Meter scores and total MIND diet score is r=0.3, *p* <0.05.

**Conclusion:**

Mean observed MIND diet and Veggie Meter scores show less than desirable intake of carotenoid‐rich foods for reduction of AD/ADRD risk among participants. The majority of participants take dietary supplements, and few consume a diet supportive of brain health. Few cohort studies include subjective and objective diet assessments with actionable feedback for participants. These preliminary findings emphasize the need for further research into dietary interventions for prevention or delay of cognitive decline.

